# Supplementary material for: Lactiplantibacillus plantarum DSM20174 Attenuates the Progression of Non-Alcoholic Fatty Liver Disease by Modulating Gut Microbiota, Improving Metabolic Risk Factors, and Attenuating Adipose Inflammation
Source: Nutrients. 2022 Dec 7;14(24):5212. doi: 10.3390/nu14245212 (PMC9787191; doi:10.3390/nu14245212)
Supplement: Supplementary file 1 [file nutrients-14-05212-s001.zip › nutrients-2041266-supplementary Table S1 and S2.pdf]

Table S1: Composition of the 2014 Teckland global 14% protein rodent maintenance diet (Teklad Diet, Envigo).

MACRONUTRIENTS	%
**Crude protein**	14.3
**Fat**	4
**Carbohydrate**	48
**Crude fiber**	4.1
**Neutral detergent fiber**	18
**Ash**	4.7
**Calories from fat**	13

Information collected from Envigo.

Table S2: Composition of the High fat diet rich in lard (Teklad Diet TD.06414, Envigo).

FORMULA	g/Kg
**Casein**	265
**L-Cystine**	4
**Maltodextrin**	160
**Sucrose**	90
**Lard**	310
**Soybean oil**	30
**Cellulose**	65.5
**Mineral mix**	48
**Calcium phosphate, dibasic**	3.4
**Vitamin mix**	21
**Choline bitartrate**	3
**Calories from fat**	60%

Information collected from Envigo.
